# Lactate-Mediated Crosstalk Between Tumor Cells and Cancer-Associated Fibroblasts: Mechanisms and Therapeutic Opportunities

**DOI:** 10.3390/ijms26125583

**Published:** 2025-06-11

**Authors:** Siqi Tan, Faxiao Zhou, Xiaoming Wu

**Affiliations:** Laboratory of Molecular Genetics of Aging & Tumor, Medical School, Kunming University of Science and Technology, Chenggong Campus, 727 South Jingming Road, Kunming 650500, China; 18144246996@163.com (S.T.); 13765899802@163.com (F.Z.)

**Keywords:** lactate, lactation, CAFs, TME

## Abstract

Lactate is a key oncometabolite that plays a critical role in modulating the behavior and function of both tumor cells and tumor-associated stromal cells within the tumor microenvironment (TME). Cancer-associated fibroblasts (CAFs), as essential stromal components, engage in dynamic crosstalk with tumor cells through lactate-mediated signaling pathways. Elevated lactate levels in the TME primarily originate from metabolic reprogramming in tumor cells and CAFs. Notably, tumor-derived lactate not only promotes basement membrane remodeling and epithelial–mesenchymal transition (EMT) in CAFs but also influences their functional phenotype. Conversely, CAF-secreted lactate significantly contributes to tumor progression. Therapeutic strategies targeting lactate transport and metabolism—particularly through the inhibition of monocarboxylate transporters (MCTs) and lactate dehydrogenase (LDH)—have emerged as promising approaches in cancer treatment. This review summarizes the multifaceted roles of lactate and lactylation, elucidates the molecular mechanisms underlying lactate-mediated tumor–CAF crosstalk, and explores potential therapeutic interventions targeting lactate metabolism and CAFs.

## 1. Introduction

With further research, lactate has been found to promote crosstalk between tumor cells and cells in the TME, thereby affecting each other’s biological functions [1,2]. Lactate secretion mainly originates from metabolically reprogrammed tumor cells and stromal cells. Among them, tumor cells favor rapid energy production by glycolysis even under aerobic conditions, a process that is accompanied by the production of large amounts of lactate. This lactate secretion is considered a typical metabolic hallmark of cancer, also known as the Warburg effect [2,3]. Lactate plays a pivotal role in promoting many aspects of tumor development, migration and invasion, metastasis, angiogenesis, and drug resistance. Concomitant studies have revealed an important milestone in lactate research, the emergence of histone lysine lactylation. This is a post-translational modification at the intersection of cellular metabolism and epigenetics [4,5,6]. In 2019, Zhang et al. first reported the lactylation of histone lysine residues, providing mechanistic validation [7]. Accumulating evidence indicates that lactate modifications extend beyond histones to non-histone proteins, underscoring the broad impact of lactate and lactylation on tumor and stromal cell biology [8,9]. Lactate can be transported to various cell populations in the TME, thereby shaping the tumor microenvironment. On one hand, the interaction between lactate and immune cells inhibits cell differentiation, establishes and maintains an immunosuppressive micro environment, and enhances tumor cell drug resistance [10,11,12,13,14]. On the other hand, lactate interactions with stromal cells and tumor-associated endothelial cells enhance basement membrane (BM) remodeling, metabolic reprogramming, EMT, angiogenesis, and drug resistance [15,16,17]. Lactic acid promotes cell-to-cell interaction in the tumor microenvironment. For example, it has been shown that immune cells secrete cytokines such as TGF-β and IL-6, which can activate CAFs [9,18]. These cytokines can influence multiple signaling pathways within CAFs. For example, TGF-β can activate the Smad signaling pathway to promote CAFs’ glycolysis and lactate production. This suggests that lactate may be indirectly regulated by these immune components.

Cancer-associated fibroblasts (CAFs) are pivotal stromal cells in the TME. They not only directly promote tumorigenesis, metabolism, metastasis, and progression but also remodel the extracellular matrix and interact with immune cells [18,19,20]. CAFs primarily focus on facilitating BM remodeling, EMT, and treatment resistance [21,22]. Notably, lactate secretion is not limited to tumor cells. Metabolically reprogrammed CAFs can also produce lactate, which subsequently influences tumor proliferation, invasion, metastasis, and drug resistance [23,24]. Furthermore, CAFs themselves are regulated by lactate derived from various tumor sources and can even recruit/activate other cell types to become CAFs [25,26]. Thus, lactate serves as a critical communication bridge between tumor cells and CAFs.

Given the rapidly growing body of research on lactate-mediated crosstalk between tumor cells and CAFs, a comprehensive review and synthesis are urgently needed. This study aims to elucidate the functional roles of lactate and lactylation, with a particular focus on analyzing how tumor-derived lactate influences cancer-associated fibroblasts (CAFs) and evaluating the subsequent tumor-promoting effects mediated by CAF-secreted lactate. Furthermore, we explore potential therapeutic strategies that target lactate metabolism and CAFs, offering valuable insights for the development of novel pharmacological interventions against these critical pathways in tumor progression.

## 2. Production and Biological Functions of Lactate

Lactic acid, with the chemical formula C_3_H_6_O_3_, undergoes dissociation in aqueous solutions, yielding a hydrogen proton (H^+^) and lactate ion (C_3_H_5_O_3_^−^). Lactate was once considered to be a metabolic waste product of cellular glycolysis and was regarded as having no significant biological role [27]. However, recent studies have demonstrated that lactate functions as both a metabolic fuel and a signaling molecule, influencing the behavior of diverse cell types, including skeletal muscle cells, cardiomyocytes, neurons, stromal cells, and malignant cells [9,28]. There are two main optical isomers of lactic acid. L-lactic acid (levorotatory): The predominant form in mammals, produced abundantly by tumor cells via the Warburg effect. D-lactic acid (dextrorotatory): The optical isomer of L-lactic acid, generated primarily from methylglyoxal (MGO) through the glyoxalase pathway. Notably, D-lactic acid occurs at much lower physiological concentrations (~11–70 nM) compared to L-lactic acid [29,30].

Lactic acid production is accomplished primarily through the glycolytic pathway. Glucose, the primary cellular nutrient, can produce energy through glycolysis and mitochondrial oxidative phosphorylation. In glycolysis, cytoplasmic glucose is progressively catabolized to pyruvate by a cascade of glycolytic enzymes, simultaneously producing ATP and NADH. Pyruvate represents a metabolic branch point, linking glycolysis to oxidative phosphorylation. In the presence of oxygen, pyruvate and NADH electrons produced by glycolysis are shuttled into the mitochondrial matrix and converted to acetyl coenzyme A, which subsequently enters the tricarboxylic acid (TCA) cycle to produce energy. Conversely, during hypoxia or periods of high energy demand, pyruvate is reduced to lactate by lactate dehydrogenase (LDH). The protonation state of lactate depends on environmental pH: In acidic microenvironments (e.g., tumor stroma), it exists predominantly as protonated lactic acid (C_3_H_6_O_3_). At physiological pH (7.2–7.4), it dissociates into lactate anions (C_3_H_5_O_3_⁻) and counterions (e.g., Na⁺).

## 3. Lactylation Modification: Mechanisms, Regulation and Function

### 3.1. Mechanism of Lactylation Modification

Lactylation is a post-translational modification (PTM) of proteins, similar to other modification processes such as phosphorylation, acetylation, methylation, and ubiquitination. In 2019, Zhao et al. identified protein lysine lactylation in human and murine cells using liquid chromatography–mass spectrometry (LC/MS) [7]. Through 13C tracing, it was demonstrated that both exogenous and endogenous lactate contribute to histone lysine lactylation. Lactylation modifications in biological systems include L-lactylation and D-lactylation, termed direct and indirect lactylation, respectively. For one, the occurrence of L-lactylation (direct lactylation) requires the participation of corresponding substrates and enzymes. The process of lactylation modification depends on three essential steps: the accumulation of lactate, the formation of lactyl coenzyme A, and enzymatic transfer. Elevated lactate levels in the tumor microenvironment, ranging from 10 mM to 40 mM, provide the necessary substrate for lactylation [8,31,32]. Subsequently, lactate binds to coenzyme A (CoA) to generate lactyl coenzyme A (Lactyl-CoA), a crucial intermediate in this modification. Finally, lactyltransferases catalyze the covalent attachment of the lactyl group to target protein residues, such as lysine. Additionally, D-lactylation, a non-histone modification prevalent in highly glycolytic tissues, utilizes methylglyoxal (MGO) as its precursor. The lactoyl moiety is indirectly supplied through *S*-D-lactoylglutathione without requiring dedicated enzymes, hence termed non-enzymatic lysine lactylation [33].

### 3.2. Regulation of Lactylation

The process of lactylation is regulated by multiple enzymes, including glycolytic enzymes, lactoyltransferases, and other modifiers. As lactate is a key metabolic byproduct of the Warburg effect, all glycolysis-related factors can modulate lactylation levels. This regulation is primarily mediated by lactate dehydrogenase (LDH), which catalyzes the reversible conversion of pyruvate to lactate [34] (Table 1). LDHA preferentially catalyzes the reduction of pyruvate to lactate while regenerating NAD+, and LDHB primarily facilitates the oxidation of lactate back to pyruvate. Despite the high lactate levels characteristic of the tumor microenvironment, less than 10% of lactate undergoes further metabolic conversion, suggesting the tight regulation of lactate flux in this context. This highlights the critical regulatory role of specific enzymes in this modification process. Acetyl coenzyme A synthetase 2 (ACSS2) and succinyl CoA synthetase (GTPSCS) have been reported to regulate the formation of lactoyl coenzyme A from the binding of lactate to coenzyme A (CoA) [35,36]. According to Zhu et al., the ACSS2/KAT2A complex emerges as a central epigenetic regulator in cancer progression, where EGFR activation induces ERK-dependent phosphorylation and the nuclear translocation of ACSS2, enabling this enzymatic complex to catalyze histone lactylation. This lactyltransferase activity subsequently reprograms transcriptional networks and fosters immune evasion, thereby driving oncogenesis [36]. In gliomas, it has been demonstrated that nuclear GTPSCS is a lactyl-CoA synthetase that forms a complex with p300, promoting the production of lactyl-CoA and histone H3K18la [35]. The process of lactoyl transfer also requires the involvement of lactoyl transferases, and histone acetyltransferases (including p300, KAT8, CGN5, HBO1) and others have been shown to play a role in this process [4,37,38,39]. P300 mediates lactate-induced histone lactylation on pro-fibrotic gene promoters in macrophages, thereby enhancing their pro-fibrotic phenotype [37]. Since the concentration of lactyl-CoA in cells is approximately 1000 times lower than that of acetyl-CoA, this has sparked scientists’ enthusiasm for exploring new key enzymes involved in lactylation modification. In colorectal cancer, the depletion of KAT8 did not reduce p300 protein expression levels, indicating that KAT8 regulation of Kla is independent of p300 [38]. Interleukin-1β-dependent GCN5 (general control non-inhibitory 5) recruitment catalyzes histone H3K18 lactylation, which regulates downstream repair gene expression after myocardial infarction [4]. Also, HBO1 has been identified as a lactyltransferase that catalyzes histone H3K9 la [39]. In addition, since lactylation is a dynamically reversible post-translational modification, lactylation modifications can also be removed under specific conditions. For example, class I histone deacetylases (HDAC1-3) and silencing proteins (SIRT) are “erasers” of histone lactylation and inhibit lactylation [40,41]. Many more modifying and de-modifying enzymes remain to be discovered.

### 3.3. Functional Roles of Lactylation in Cancer

Lactylation modifications are classified into histone lactylation and non-histone lactylation based on their target proteins, each with distinct functional roles. For one, histone lysine lactylation (Kla) regulates gene expression and tumor progression. In bone-marrow-derived macrophages (BMDMs), lactate treatment increases Kla levels at the *ARG1* promoter region, upregulating *PDGFA* expression [7]. It has been well reported that histone lactylation affects tumor biological processes [42,45,46]. In particular, lysine lactylation at position 18 of histone H3 promotes tumorigenesis [47,48], immune escape [49], and drug resistance [50]. In addition, Kla induces the expression of TCA-cycle enzymes. In breast cancer, CAF-derived lactate enhances zinc-finger protein 64 (ZFP64) histone lactylation, conferring adriamycin (DOX) resistance [51]. Emerging evidence highlights lactylation’s role in non-histone proteins. Non-histone lactylation can also promote tumor growth, invasion and metastasis, and immune escape [52,53]. A growing number of studies have reported that the lactylation of various enzymes can affect the biological functions of different tumor cells. For example, Zong et al. found that in colorectal cancer, alanyl-tRNA synthetase 1 (AARS1) promoted the lactylation of p53 and inhibited p53 phase separation, DNA binding, and transcriptional activation [54]. In gastric cancer, AARS1 acts as a lactoyltransferase, shuttling lactate to the nucleus to activate YAP, forming a YAP-TEAD-driven feedback loop [55]. Yang et al. found that lactate also leads to the lactylation of adenylate kinase 2 (AK2) K28, which enhances the proliferation and metastasis of HCC cells [56]. MCT plays an important role in the lactate shuttling between cells. In prostate cancer, MCT1 mediates lactate uptake into PCa cells and lactate-activated HIFα, thereby promoting prostate cancer angiogenesis [57]. In addition to MCT, lactic acid can also bind to G-protein-coupled receptor 81 (GPR81) to promote tumor cell energy metabolism. GPR81 promotes the proliferation and metastasis of pancreatic cancer by facilitating the transport of lactate, while knocking down GPR81 leads to a decrease in mitochondrial activity and tumor cell death [58]. Moreover, lactylation can also affect chemoresistance, such as the lactylation of key components of the gene repair complex MRN, which enhances DNA repair and induces chemoresistance [59,60]. In summary, lactate and lactylation critically regulate tumor survival, proliferation, and metastasis, but the different roles of lactation in different tumors remain to be explored.

## 4. Lactate-Mediated Crosstalk Between CAFs and Tumors

### 4.1. Lactate Secretion and Shuttling in CAFs

**Figure 1:**
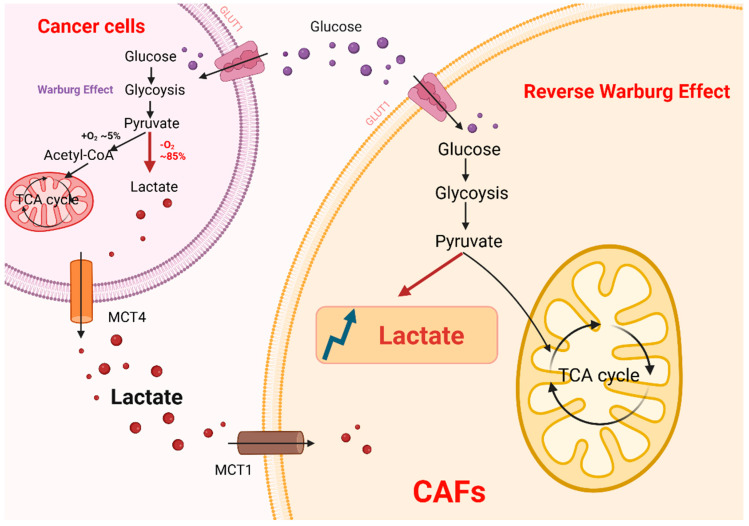
Metabolic interactions between tumor cells and cancer-associated fibroblasts (CAFs). Tumor cells take in a lot of glucose through glycolysis and turn it into pyruvate. Under the Warburg effect, pyruvate is mainly converted into lactate instead of going into the mitochondrial TCA cycle. Lactate is secreted into the tumor microenvironment through monocarboxylate transporters (MCT4). Lactic acid signaling induces metabolic reprogramming in CAFs, activating the reverse Warburg effect, whereby CAFs enhance glycolysis and further secrete lactic acid.

The metabolic symbiosis between cancer-associated fibroblasts (CAFs) and tumor cells orchestrates CAF-driven lactate secretion [61,62] (Figure 1). While tumor cells rely on the Warburg effect to sustain rapid proliferation, peritumoral CAFs exhibit a parallel glycolytic phenotype termed the “reverse Warburg effect” [63,64]. The two-compartment metabolic model functions through CAF activation and metabolic coupling, wherein tumor-derived signals such as HIF-1α, TGF-β, and ROS induce stromal autophagy and CAF differentiation, leading to aerobic glycolysis in CAFs that produce lactate, pyruvate, fatty acids, and ketone bodies as metabolic substrates for tumors. These metabolites, particularly lactate, are shuttled between CAFs and tumor cells via monocarboxylate transporters (MCTs 1-4), a subclass of solute carriers belonging to the SLC16A family, thereby sustaining tumor growth through metabolic symbiosis. MCT belongs to the solute carrier (SLC) family, which consists of fourteen members, with the first four isoform members present in humans involved in lactate shuttling in the tumor microenvironment. The MCT family enables lactate exchange across the plasma membrane via H+/lactate cotransport in the direction of the plasma membrane, which is dependent on the plasmonic and monocarboxylate concentration gradient [65]. In hypoxic tumor cells, lactate production is driven by LDHA-mediated glycolysis, with subsequent MCT4-dependent lactate extrusion. Conversely, oxidatively stressed CAFs exhibit aerobic glycolysis (reverse Warburg effect), generating metabolic fuels (lactate, pyruvate, fatty acids, and ketones) to support tumor growth. Normoxic tumor cells can take up “fuels” (e.g., lactate) produced by CAFs via MCT1. After uptake, lactate is converted to pyruvate by LDHB, which is subsequently oxidatively phosphorylated (OXPHOS) in mitochondria to produce large amounts of ATP for the rapid proliferation of tumor cells [66]. Critically, the resulting lactate accumulation acidifies the tumor microenvironment, promoting therapy resistance in both tumor cells and CAFs [67].

### 4.2. Tumor-Promoting Effects of CAF-Derived Lactate

Lactate produced by CAFs via the reverse Warburg effect promotes tumor development, angiogenesis, drug resistance, and many other effects [20,57,59,60]. Numerous studies have revealed and summarized the functions of CAFs in promoting different tumors through various secreted factors and shaping the extracellular matrix [68,69]. The roles of lactic acid secreted by CAFs in different cancers, such as oral, prostate, and breast cancers, are yet to be summarized [70].

**Figure 2:**
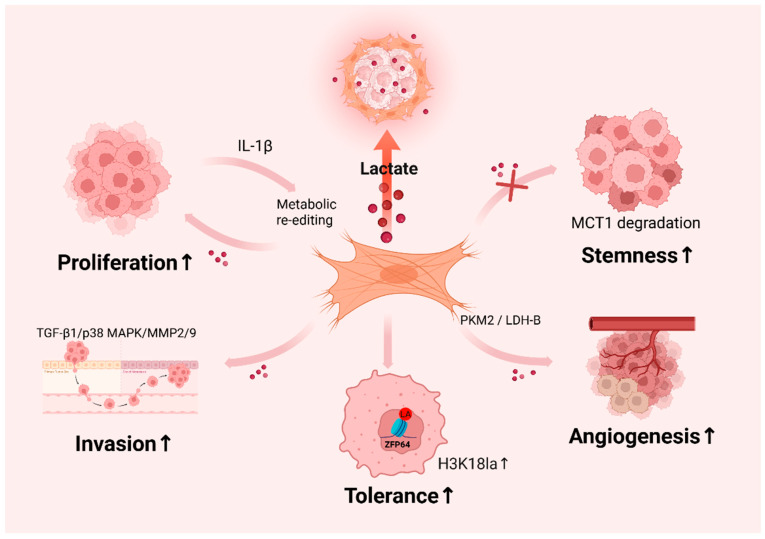
Lactic acid secreted by CAFs promotes tumor development. Lactate secreted by cancer-associated fibroblasts (CAFs) has multiple pro-cancerous effects in the tumor microenvironment, including promoting tumor proliferation, invasion, stemness maintenance, and immune evasion.

The efficiency and degree to which different tumor cells utilize lactate varies, enabling tumor cells to effectively utilize lactate to promote their own growth [71]. For example, GPR81 is highly expressed in breast cancer cells, and extracellular lactate binding to GPR81 activates the phosphoinositide 3-kinase (PI3K)/protein kinase B (Akt) pathway, promoting tumor angiogenesis [72]. Furthermore, high-density ECE in tumors restricts the diffusion of lactic acid, resulting in increased local concentrations and promoting tumor growth [73,74]. This gives lactic acid a heterogeneous effect on different tumors. Zhang and team members observed that OSCC cells (oral cancer cells) enhance stromal glycolysis in activated fibroblasts by partitioning IL-1β, leading to the up-regulation of MCT4 (a lactate efflux transporter protein) in CAFs to promote lactate uptake and proliferation in OSCC [75]. Fischi et al. observed that human prostate cancer (PCa) cells induce a high expression of GLUT1 and MCT4 in CAF cells to increase glucose uptake and lactate export in CAF. In contrast, PCa cells were reprogrammed upon contact with CAFs to utilize the lactate produced by CAFs to support their IL-1β tricarboxylic acid (TCA) cycle, anabolic processes, and cell proliferation [76]. At the same time, the lactate secreted by these CAFs promotes tumor invasion. Lactate derived from hypoxic CAFs provides a metabolic coupling between CAFs and breast cancer cells and promotes breast cancer cell invasion by activating the TGF-β1/p38 MAPK/MMP2/9 signaling axis and facilitating mitochondrial activity in the cancer cells [24]. Lactate secreted by CAFs also promotes angiogenesis, which further strengthens the malignancy of the tumor. In breast cancer, CAFs promote tumor growth and angiogenesis by providing energy-rich metabolites (e.g., L-lactic acid) in a paracrine manner [77]. CAF-derived lactic acid also enhances cancer stemness by inhibiting the ubiquitinated degradation of MST1 in OSCC [78]. In addition, it has recently been shown that lactic acid secreted by CAFs upregulates tumor cell histone lactylation and exerts a pro-tumorigenic function. CAFs-derived lactic acid regulates iron death by enhancing histone H3K18la lactylation of ZFP64, which promotes drug resistance in breast cancer cells [51]. 2025 Li T et al. reported perineurial nerve infiltration (PNI) related cancer-associated fibroblasts (pCAFs) with high levels of glycolysis can form a high-lactic acid tumor microenvironment that favors pancreatic cancer. pCAFs secrete lactic acid that promotes the lactylation of histone H3K18 in tumor cells, an epigenetic modification that upregulates the neuroinvasion-associated genes L1CAM and SLIT to enhance tumor invasive capacity [79]. Thus, CAFs-derived lactate plays an important role in promoting tumor growth and proliferation, invasion, angiogenesis, tumor stemness, and drug resistance (Figure 2).

### 4.3. Lactate-Induced CAF Activation

**Figure 3:**
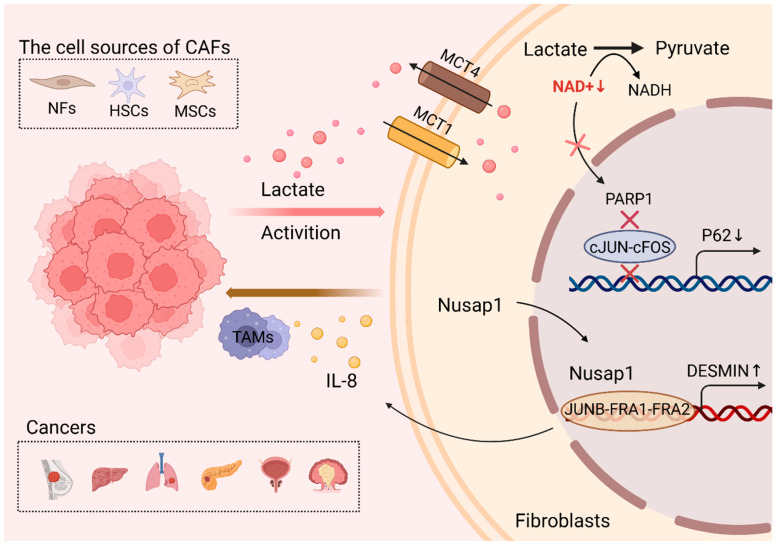
Tumor-secreted lactic acid activates CAFs.

Meanwhile, lactate serves as a key signaling molecule in the activation of CAFs [70]. Most CAFs originate from resident fibroblasts, and can also be transformed from adipocytes, pericytes, endothelial cells, and bone-marrow-derived macrophages. In order to accurately identify this heterogeneous group of CAFs, common markers of CAFs include alpha smooth muscle actin (α-SMA), fibroblast activation protein (FAP), fibroblast specific protein (FSP) and vimentin, etc. [79]. Increasingly, studies have shown that lactic acid activates activated CAFs in a novel way in cancers such as breast, liver, lung, and pancreatic ductal carcinoma (Figure 3).

Several studies have shown that lactate secreted by different cancer cells activates and recruits CAFs. In breast cancer, POU1F1 transcription factor in breast cancer cells enhances LDHA expression and promotes pro-lactate and secretion into the TAM. Normal fibroblasts (NFs) take up lactate and upregulate the expression of a-SMA at the transcriptional and protein levels and convert it into CAFs [80]. Similarly, in hepatocellular carcinoma (HCC), hypoxia-induced integrin β4 (ITGB4) activates the TGF-β/Smad pathway, promoting lactate secretion that transforms hepatic stellate cells (HSCs) into CAFs marked by elevated α-SMA and vimentin expression [81]. The molecular mechanisms underlying lactate-mediated CAF activation are increasingly being elucidated. In lung cancer, lactate induces the nuclear translocation of nucleolar and spindle-associated protein 1 (NUSAP1) in fibroblasts, which facilitates JUNB-FRA1-FRA2 transcriptional complex binding to the DESMIN promoter, ultimately upregulating CAF markers (DESMIN, TGFB1, SDF1) [26]. Meanwhile, lactate-activated CAFs secrete IL-8, which in turn upregulates the immune checkpoint protein PD-L1 and TAM s markers to promote immune infiltration [26]. Pancreatic ductal adenocarcinoma (PDAC) studies reveal that lactate induces epigenetic reprogramming of mesenchymal stem cells (MSCs) via α-ketoglutarate (αKG)-dependent TET enzyme activation, leading to global DNA demethylation (reduced 5 mC) and hydroxymethylation (increased 5 hmC) that drives CAF differentiation [82]. In prostate cancer, NFs take up lactic acid secreted by prostate cancer, deactivate ADP-ribose polymerase 1 (PARP-1), leading to the downregulation of p62 in stromal cells, and activate CAFs by upregulating the expression of CAF markers SDF1, HA, and a-SMA at the transcriptional and protein levels [25]. Notably, histone lactylation in CAFs has emerged as a critical regulator of tumor progression. In lung cancer, lactate promotes the pan-lysine lactylation (pan-Kla) and H3K18la levels of CTHRC1 + CAF, thereby promoting resistance to EGFR-TKI [83]. Multi-omics analyses in clear-cell renal cell carcinoma identified TIMP1 as a lactylation-regulated gene predominantly expressed in myofibroblastic CAFs (myoCAFs) [84]. This finding is corroborated by lung cancer studies showing histone lactylation in infiltrating myoCAFs [84]. Recent single-cell RNA sequencing data further demonstrate that colorectal cancer-derived lactate converts normal fibroblasts to CAFs, evidenced by upregulated ACTA2, vimentin, and COL1A1 expression, along with enhanced proliferative, migratory, and invasive capacities [85]. Studies on the molecular mechanisms of the lactate-induced activation of CAFs secreted in different tumors need to be further investigated.

Lactic acid secreted by different tumor cells can activate three types of precursor cells into CAFs: normal fibroblasts (NFs), hepatic stellate cells (HSCs), and mesenchymal stem cells (MSCs). The phenomenon of lactic acid activation has been reported in many types of tumors, including breast cancer, liver cancer, lung cancer, pancreatic ductal adenocarcinoma, prostate cancer, and colorectal cancer.

## 5. Therapeutic Strategies Targeting Lactate in Cancer

Lactate serves as a critical metabolic bridge between tumor cells and CAFs, making it a promising therapeutic target for simultaneously disrupting both CAFs and tumor cells. However, the bidirectional lactate shuttle between CAFs and tumor cells complicates efforts to effectively target CAFs. CAFs also directly secrete factors and deliver exosomes to enhance tumor resistance, making it challenging to target CAFs in studies targeting stromal cells to treat tumors [68,86] (Table 2).

Emerging evidence highlights lactate as a key mediator of therapy resistance. It has been shown that non-small-cell lung cancer cell (NSCLC)-derived lactate is a key molecule in tumor resistance to tyrosine kinase inhibitors (TKIs). Lactate secreted by lung cancer promotes the production of hepatocyte growth factor (HGF) by CAFs in an NF-κB-dependent manner, which activates MET-dependent signaling in cancer cells and sustains resistance to TKIs [15]. Beyond stromal interactions, lactate also induces immunosuppression, impairing the efficacy of anticancer therapies [101,102]. For instance, in pancreatic cancer, radiotherapy upregulates glycolysis and lactate secretion, which recruits myeloid-derived suppressor cells (MDSCs) and fosters an immunosuppressive tumor microenvironment. Elevated protein lactylation further reinforces this immunosuppressive phenotype, contributing to treatment resistance in pancreatic ductal adenocarcin [103]. Moreover, lactate accumulation results in an acidic environment that impairs immune cell function and promotes tumor growth [67]. Lactate accumulation significantly reduces the extracellular PH to 6.0–6.5, well below the normal range of 7.3–7.4. This pH change leads to the inactivation of CD8^+^ T cell nuclear NK cells, while enhancing the immunosuppressive properties of immunosuppressive cells such as regulatory T cells, Tregs. Mechanistically, lactate suppresses antitumor immunity through multiple pathways. Lactate promotes GPR81 receptor internalization in lung tumor cells, driving programmed death-ligand 1 (PD-L1) expression and thereby inhibiting CD8+ T cell-mediated tumor killing [104]. Secondly, lactate upregulates chemokines such as CXCL12 and CX3CL1, which recruit Tregs and tumor-associated macrophages (TAMs), further reinforcing an immunosuppressive niche [14,105]. Given lactate’s dual role in fostering CAF-tumor crosstalk and immune evasion, there is a pressing need to review therapeutic strategies targeting lactate metabolism and its interplay with CAFs.

### 5.1. Targeting Monocarboxylate Transporters (MCTs)

Lactate transport plays a key role in the metabolic re-editing of tumor cells and CAFs and is mainly mediated by MCT1 and MCT4 in the MCT family. MCT1 facilitates lactate uptake while MCT4 mediates lactate efflux, collectively promoting tumor progression and CAF activation [106,107]. To inhibit lactate uptake, researchers have investigated a series of MCT inhibitors. Inhibitors targeting MCT1, including AZD3965, have been found to inhibit intracellular proliferation of a variety of tumors, including lung cancer [87], breast cancer [88,108], and lymphoma [89,109,110]. Preclinical studies reveal that AZD3965 not only alters tumor cell metabolism but also enhances infiltration of antitumor immune cells, effectively suppressing tumor growth in murine models. Notably, AZD3965 has progressed to Phase I/II clinical trials (NCT01791595), showing promising results in advanced solid tumors and non-Hodgkin’s lymphoma [87,109,111]. However, the compensatory upregulation of MCT4 often limits the efficacy of single-agent MCT1 inhibition. Research on small-cell lung cancer has found that knocking down MCT4 is necessary to make tumors sensitive to the MCT1 inhibitor AZD396, indicating that the compensatory effect of MCT4 inhibits the antitumor effect of MCT1 inhibitors [87]. To address this issue, a growing number of studies have explored dual MCT1/MCT4 inhibition [90,112]. Recent studies have shown that MCT inhibition does not reduce the release of lactic acid from cells. This may be due to the accumulation of toxic lactic acid in the cytoplasm or off-target effects leading to tumour death, rather than blocking glycolysis itself [107,113]. The combination can target the lactate shuttle with greater antitumor capacity. For instance, combining AZD3965 with immune checkpoint inhibitors (ICIs) synergistically reverses lactate-mediated immunosuppression by restoring immune cell function within the tumor microenvironment [90].

Given lactate’s dual role in promoting tumorigenesis and CAF activation, MCT inhibitors represent a promising strategy for simultaneously targeting both tumor cells and CAFs. Emerging evidence suggests that combining MCT inhibition with metabolic modulators like metformin can further amplify therapeutic effects [114]. Metformin (1,1-dimethylbiguanide), a well-tolerated and cost-effective antidiabetic drug, has demonstrated significant antitumor activity in both preclinical and clinical settings across various malignancies (e.g., lung adenocarcinoma, ovarian cancer, breast cancer) [115,116,117,118]. Mechanistically, metformin disrupts metabolic crosstalk between tumors and CAFs [115], and specifically inhibits HIF-1α-driven SDF1-mediated pro-tumorigenic signaling in CAFs [118]. These properties position metformin as both a potential therapeutic agent and biomarker for targeting lactate metabolism and CAF activity in cancer treatment.

### 5.2. Lactate Dehydrogenase (LDH) Inhibitors and Nanoparticle Delivery

Lactate dehydrogenase (LDH)-targeted therapies have emerged as promising strategies for cancer treatment, demonstrating significant potential in preclinical and clinical models [119,120,121]. Among the LDH isoforms, LDHA plays a pivotal role in tumor metabolism by catalyzing the conversion of pyruvate to lactate, thereby sustaining glycolysis through NAD+ regeneration while simultaneously acidifying the tumor microenvironment (TME). This metabolic reprogramming promotes tumor progression, immune evasion, and therapeutic resistance [122,123,124]. Consequently, substantial efforts have been directed toward developing and validating LDHA inhibitors as anticancer agents [119,125].

Among them, Oxamate is a competitive LDHA inhibitor that binds to the pyruvate active site, blocking lactate production. In gastric, lymphoma, pancreatic, and hepatocellular carcinoma tumors, Oxamate exerts the ability to inhibit lactic acid production, reduce TME acidity, and inhibit tumors [91,92,93,94]. In hepatocellular carcinoma, Oxamate synergizes with PD-1 inhibitors by suppressing B7-H3 upregulation, a mechanism mediated through the inhibition of histone H3K18 lactylation, thereby enhancing antitumor immunity [94]. FX11, a potent LDHA inhibitor, exhibits significant antitumor activity against pancreatic cancer, neuroblastoma, thyroid cancer, and lymphomas [96,126]. FX11 is also used in conjunction with other drugs to enhance anticancer activity, e.g., the combination of FX11 with FK866, an inhibitor of NAD^+^ synthesis, inhibits tumor growth through dual metabolic blockade [95,96]. In pancreatic cancer, the combination of metformin and FX11 reduced the lactate level of PANC-1 cells through the AMPKa axis, which led to an increase in cancer cell stress, leading to apoptosis [97]. Additionally, FX11 decreases CAF abundance in pancreatic tumors, alleviating fibrosis and immunosuppression in the TME. Nanoparticles targeting the lactate dehydrogenase LDHA are safer and more efficient therapeutic modalities, including nanocarriers containing LDHA inhibitors and nanoparticles silencing and editing the LDHA gene [86,99]. LDHB is a participant in the lysosomal activity and autophagy of cancer cells, and participates in the process of self-promotion of tumorigenicity and the development of drug resistance in cancer cells [127]. Thus, LDHB may be a promising target for cancer prevention and treatment. It has been reported that the selective knockdown of LDHB in breast cancer significantly reduces the proliferation of cancer cells in vitro and in vivo [128]. Recently, Sachio Shibata et al. identified a chemical probe, AXKO-0046, that selectively inhibits LDHB activity affecting cancer metabolism. Nanoparticle delivery systems also show strong potential in targeting CAFs. Lactate secreted by tumor cells and CAFs impairs the function of tumor immune cells and promotes the recruitment of immunosuppressive cells such as M2 macrophages and Tregs [100]. This multifaceted strategy holds significant promise for overcoming the therapeutic challenges posed by metabolic symbiosis between tumors and their microenvironment.

## 6. Discussion

Current understanding of lactate-mediated interactions between tumor cells and cancer-associated fibroblasts (CAFs) remains limited. This review synthesizes current knowledge on lactate’s dual role as a metabolic substrate and a signaling molecule, highlighting its impact on CAF activation, tumor–stromal interactions, and therapeutic resistance. Despite advances, several unresolved questions and translational challenges persist.

### 6.1. Lactylation: A Frontier in Epigenetic and Metabolic Regulation

The discovery of lactylation as a post-translational modification (PTM) has revolutionized our view of lactate’s functionality, positioning it as a direct modulator of gene expression and protein activity. Histone lactylation (e.g., H3K18la) drives pro-tumorigenic gene programs, such as *PDGFA* upregulation in macrophages and *ZFP64*-mediated chemoresistance in breast cancer [51]. Non-histone lactylation (e.g., p53, AK2) further expands lactate’s regulatory scope, influencing DNA repair, metastasis, and immune evasion. However, key gaps remain: (1) The identification of lactate modification-related enzymes is still imperfect. The specific transferase and demodifying enzymes in the process of lactate modification have not yet been clarified, which greatly limits the in-depth understanding of the regulatory mechanism of lactate modification. (2) Functional consequences: Does lactylation confer novel protein functions beyond modulating existing pathways? For instance, lactylated MRN complex components enhance DNA repair [59], but whether this creates gain-of-function phenotypes is unclear. (3) A lack of tools to regulate lactylation levels. Currently, the commonly used methods to regulate lactylation include the point mutation of lactylation sites, treatment with foreign lactic acid, the inhibition of upstream regulators of glycolysis, and orthogonal translation systems using pyrrolidyl-tRNA synthetase. However, the lactylation levels of the above methods cannot be precisely controlled and do not reflect the true pathological conditions. (4) Challenges exist in developing effective lactate/lactylation-targeted therapies.

### 6.2. Heterogeneity of CAFs and Lactate-Driven Phenotypes

Several reports have revealed that lactate can activate CAFs, but the molecular mechanisms of how lactate and lactylation regulate the expression of markers of CAFs and affect the physiological functions of CAFs are not known. Recent single-cell studies reveal lactylation-associated CAF subpopulations in renal and colorectal cancers [84,85]. For example, TIMP1^+^ myCAFs in renal carcinoma exhibit lactylation-dependent pro-angiogenic signatures [85]. An in-depth study of the effects of lactate and lactylation on CAFs faces the following questions: (1) Whether lactate and lactylation affect the heterogeneity of CAFs. The activation of normal cells into CAFs is accompanied by the acquisition of some common markers and tumor-promoting capacity. Different markers have been used to identify different subtypes of CAFs, such as myCAF and iCAF. However, due to the numerous markers of CAFs, an in-depth study of lactate and lactylation may identify different CAFs in a novel way. (2) The development of synergistic therapeutic strategies that simultaneously target lactate metabolism and CAFs still requires multidisciplinary research and technological innovation. Except for metformin and LDHA nanoparticles, which have the potential to target both lactate and CAFs, there is still a long way to go in the study of targeting both at the same time. (3) The regulatory mechanism of lactic acid and lactation on the secretion function of CAFs is unknown. A 2024 article reported that lactic acid in lung cancer can activate CAFs and enhance the secretion of IL-8 by CAFs [26], but the study of lactic acid and lactation on the secretion process of CAFs needs to be deepened.

In conclusion, lactate is a linchpin of tumor–stromal crosstalk. While therapeutic targeting is nascent, integrating lactate modulation with stromal and immune-directed strategies offers a transformative approach to cancer treatment.

## Figures and Tables

**Table 1:** Regulatory mechanisms of lactate modification processes.

Processes	Enzyme	Mechanism	References
Lactic acid accumulation	LDHA, LDHB	LDHA preferentially catalyzes the reduction of pyruvate to lactate while regenerating NAD+, and LDHB primarily facilitates the oxidation of lactate back to pyruvate.	[34]
The formation of lactyl coenzyme A	ACSS2	The ACSS2/KAT2A complex emerges as a central epigenetic regulator in cancer progression, where EGFR activation induces ERK-dependent phosphorylation and the nuclear translocation of ACSS2.	[36]
	GTPSCS	Nuclear GTPSCS functions as a lactyl-CoA synthetase that complexes with p300 to promote lactyl-CoA production and subsequent histone H3K18 lactylation (H3K18la).	[35]
Lactyltransferase	p300	P300 mediates lactate-induced histone lactylation on pro-fibrotic gene promoters in macrophages.	[37,42]
	KAT8	KAT8 identified as eEF1A2 K408 lactate transferase, promoting colorectal tumor growth.	[36,38]
	CGN5	Interleukin-1β-dependent GCN5 (general control non-inhibitory 5) recruitment catalyzes histone H3K18 lactylation.	[4]
	HBO1	HBO1-mediated H3K9la has been confirmed to be associated with tumorigenesis across multiple cancer cell lines, including HeLa (cervical cancer), HepG2 (hepatocellular carcinoma), U87MG (glioblastoma), KYSE-30 (esophageal squamous cell carcinoma), MDA-MB-231 (breast cancer), HCT116 (colon cancer), and H460 (non-small cell lung cancer).	[39]
De-lactylation modification	HDAC1-3	HDAC1-3 exhibits potent activity not only against K(L-la) but also against K(D-la) and various short-chain acyl modifications. HDAC1 and HDAC2: reversing H3K9la, H3K18la, and H4K8la.	[40]
	SIRT	SIRT2 can remove the lactyl group from synthetic peptides related to pyruvate kinase M2 (PKM2), SIRT3 exhibits class-selective histone deacetylase activity, preferentially recognizing H3 K4, K9, K18, K23, K27, and H4K16.	[41,43,44]

**Table 2:** Relevant lactate-targeting agents.

	The Main Targeted Drugs	Cancer Types and CAFs	The Study Outcomes	Ref	Limitation
MCT inhibitors	AZD3965	Small Cell Lung cancer	Patients who express MCT1 but not MCT4 have a better outcome.	[87]	The compensatory function of MCT4
	AZD3965	Breast cancer	AZD3965 exerts slowly reversible inhibition of MCT1-mediated L-lactate uptake.	[88]	
	AZD3965 and OXPHOS	Lymphoma	Combining AZD3965 with an inhibitor of oxidative phosphorylation (OXPHOS) can induce significant tumor cell death.	[89]	
	AZD3965,a novel MCT4 inhibitor and immune checkpoint drugs NAC and AZD3965	Colorectal cancer Colorectal cancer and CAFs	Improved leukocyte infiltration and T cell activation, delayed tumor growth, and prolonged survival in vivo. It inhibits the expression of NF-κB and HIF-1α, thereby exerting its anticancer function.	[90] [23]	
LDH inhibitors and nanoparticle delivery systems	Oxamate Oxamate Oxamate Oxamate and PD-1 inhibitor FX11 and FK866 FX11 and metformin FX11 AXKO-0046 Nanoparticles editing the LDHA gene	Colorectal cancer Nasopharyngeal carcinoma Gastric cancer Hepatocellular Breast cancer Pancreatic cancer CAFs and PC - Cancers	Triple therapy abolishes proliferation and induces apoptosis and autophagy in CRC cells through the induction of ULK1 which is regulated by the mir-26a/HIF-1α axis Oxamate increased the radiosensitivity in NPC cells in vitro Oxamate-mediated inhibition of the Akt-mTOR signaling pathway activates autophagy and exhibits anticancer activity. Through inhibition of histone H3K18 lactylation, thereby enhancing antitumor immunity. Inhibits tumor growth through dual metabolic blockade. Activation of the AMPKα axis causes stress and apoptosis in tumor cells.Reduces the number of CAFs. A chemical probe that inhibits LDHB. Inhibition of glycolysis in multiple tumor cells.	[91] [92] [93] [94] [95,96] [97] [86] [98] [99,100]	Inhibit the normal function of LDH The compensatory mechanisms of metabolic pathways

## Abbreviations

**Abbreviation**	**Full name**
ACSS2	Acetyl-CoA synthetase 2
AK2	Adenylate kinase 2
α-SMA	Alpha smooth muscle actin
αKG	Alpha-ketoglutarate
ARG1	Arginase 1
AARS1	Alanyl-tRNA synthetase 1
ATP	Adenosine triphosphate
BMDMs	Bone-marrow-derived macrophages
BM	Basement membrane
CAFs	Cancer-associated fibroblasts
COL1A1	Collagen type I alpha 1 chain
CXCL12/SDF1	C-X-C motif chemokine ligand 12
CX3CL1	C-X3-C motif chemokine ligand 1
D-LA	D-lactic acid
ECM	Extracellular matrix
EMT	Epithelial–mesenchymal transition
FAP	Fibroblast activation protein
FSP	Fibroblast-specific protein
GLUT1	Glucose transporter 1
GPR81	G protein-coupled receptor 81
GTPSCS	GTP-specific succinyl-CoA synthetase
H3K9la	Histone H3 lysine 9 lactylation
H3K18la	Histone H3 lysine 18 lactylation
H4K8la	Histone H4 lysine 8 lactylation
HBO1	Histone acetyltransferase binding to ORC1
HCC	Hepatocellular carcinoma
HDAC1-3	Histone deacetylase 1-3
HIF-1α	Hypoxia-inducible factor 1-alpha
HSCs	Hepatic stellate cells
IL-1β	Interleukin-1 beta
IL-8	Interleukin-8
iCAF	Inflammatory cancer-associated fibroblast
ITGB4	Integrin beta 4
KAT2A	Lysine acetyltransferase 2A
KAT8	Lysine acetyltransferase 8
Kla	Lysine lactylation
L-LA	L-lactic acid
LDHA	Lactate dehydrogenase A
LDHB	Lactate dehydrogenase B
MCT1-4	Monocarboxylate transporter 1-4
MDSCs	Myeloid-derived suppressor cells
myoCAF	Myofibroblastic cancer-associated fibroblast
NAD+	Nicotinamide adenine dinucleotide
NFs	Normal fibroblasts
OXPHOS	Oxidative phosphorylation
TCA	Tricarboxylic acid cycle
TME	Tumor microenvironment
YAP	Yes-associated protein
ZFP64	Zinc-finger protein 64
